# Antibiotic Susceptibility and Molecular Characterization of Uropathogenic *Escherichia coli* Associated with Community-Acquired Urinary Tract Infections in Urban and Rural Settings in South Africa

**DOI:** 10.3390/tropicalmed5040176

**Published:** 2020-11-27

**Authors:** Purity Z. Kubone, Koleka P. Mlisana, Usha Govinden, Akebe Luther King Abia, Sabiha Y. Essack

**Affiliations:** 1 Antimicrobial Research Unit, College of Health Sciences, University of KwaZulu-Natal, Durban 4000, South Africa; zamapk@yahoo.com (P.Z.K.); govindenu@ukzn.ac.za (U.G.); essacks@ukzn.ac.za (S.Y.E.); 2 Department of Medical Microbiology, School of Laboratory Medicine and Medical Sciences, University of KwaZulu-Natal, Durban 4000, South Africa; mlisanak@ukzn.ac.za

**Keywords:** community-acquired, urinary tract infections, extended-spectrum beta-lactamases, antibiotic resistance, clonal relatedness, uropathogenic *E. coli*, treatment options, multidrug resistance, antibiotic resistance genes, fluoroquinolones, rural and urban communities

## Abstract

We investigated the phenotypic and genotypic antibiotic resistance, and clonality of uropathogenic *Escherichia coli* (UPEC) implicated in community-acquired urinary tract infections (CA-UTIs) in KwaZulu-Natal, South Africa. Mid-stream urine samples (*n* = 143) were cultured on selective media. Isolates were identified using the API 20E kit and their susceptibility to 17 antibiotics tested using the disk diffusion method. Extended-spectrum β-lactamases (ESBLs) were detected using ROSCO kits. Polymerase chain reaction (PCR) was used to detect uropathogenic *E. coli* (targeting the *pap*C gene), and β-lactam (*bla*_TEM_*/bla*_SHV_-like and *bla*_CTX-M_) and fluoroquinolone (q*nr*A, q*nr*B, q*nr*S, *gyr*A, *par*C, a*ac(6’)-Ib-cr*, and q*ep*A) resistance genes. Clonality was ascertained using ERIC-PCR. The prevalence of UTIs of Gram-negative etiology among adults 18–60 years of age in the uMgungundlovu District was 19.6%. Twenty-six *E. coli* isolates were obtained from 28 positive UTI samples. All *E. coli* isolates were *pap*C-positive. The highest resistance was to ampicillin (76.9%) and the lowest (7.7%) to amoxicillin/clavulanic acid and gentamycin. Four isolates were multidrug-resistant and three were ESBL-positive, all being *CTX-M*-positive but *SHV*-negative. The a*ac(6’)-Ib-cr* and *gyr*A were the most detected fluoroquinolone resistance genes (75%). Isolates were clonally distinct, suggesting the spread of genetically diverse UPEC clones within the three communities. This study highlights the spread of genetically diverse antibiotic-resistant CA-UTI aetiologic agents, including multidrug-resistant ones, and suggests a revision of current treatment options for CA-UTIs in rural and urban settings.

## 1. Introduction

Urinary tract infections (UTIs) are among the most common bacterial infections globally. By 2016, it was estimated that 150 million cases occurred annually worldwide [1]. These infections are a substantial cause of morbidity in both males and females with the latter being more frequently infected [1]. UTIs can be hospital-acquired (HA) or community-acquired (CA) [2]. CA-UTIs are defined as those UTIs occurring within a community or within 48 hours of hospitalization and not incubating at the time of hospitalization [3]. These infections are the second frequently diagnosed infections in communities [4].

Many different bacteria have been identified as the aetiologic agents of UTIs, some of which include *Pseudomonas aeruginosa*, *Proteus vulgaris*, *Proteus mirabilis*, *Enterobacter cloaca*, *Enterobacter aerogenes*, *Staphylococcus aureus*, *Escherichia coli*, and *Klebsiella pneumoniae* [5,6]. The most common uropathogens are *Escherichia coli* and *Klebsiella pneumonia*, which are also the UTI indicator organisms in the Global Antimicrobial Surveillance System (GLASS) of the World Health Organization [7,8]. However, over 80% of all uncomplicated CA-UTIs are caused by *E. coli* alone [9]. Although usually considered human commensals, some *E. coli* pathotypes such as the uropathogenic *E. coli* (UPEC) have developed abilities that allow them to breach the normally sterile urinary tract, causing both symptomatic and asymptomatic infections, especially in immunocompromised individuals [9]. 

Although different guidelines for the treatment of UTIs, based on extensive scientific evidence, have been adopted in different parts of the world [9], many of the causative agents of UTIs have become resistant to most antibiotics used for their treatment, presenting an enormous challenge to the use of these empirical treatment options [10]. Increasingly more UTIs are caused by multidrug-resistant organisms, including *E. coli*, worldwide [11]. This emergence of multidrug-resistant isolates has further complicated the efficacy of antibiotics, reducing therapeutic options in the health services, and subsequently increasing medical costs, morbidity, and mortality rates [12]. Even more demanding is the fact that the susceptibility profile of uropathogenic bacteria varies depending on the type of healthcare facility, geographic location, environment, and, the period of evaluation of susceptibility profiles of pathogens [1]. 

It is suggested that for effective UTI treatment to be achieved, it is imperative to acquire knowledge of the antibiograms of the causative agents [10]. Thus, the present study describes the molecular epidemiology of *E. coli* from a point prevalence study conducted among outpatients presenting with UTIs at three community health centers and an out-patient department of a district hospital in uMgungundlovu District, KwaZulu-Natal, South Africa.

## 2. Materials and Methods 

### 2.1. Ethical Considerations

Ethical approval was obtained from the Biomedical Research Ethics Committee (BREC) (Reference number: BF 515/16, sub-study of BCA444/16), and the KwaZulu-Natal Provincial Health Ethics Committee (NHRD Reference: 2016_RP57_394). Permission to conduct the research was further obtained from the KwaZulu-Natal Department of Health, the uMgungundlovu District Manager and health facility managers.

### 2.2. Study Design and Study Sites

This study was a point prevalence study, in which the number of individuals with a disease in a time interval is divided by the total number of individuals in a population [13]. The study examined CA-UTIs at three community health centers (CHCs) in the uMgungundlovu District, KwaZulu-Natal on a single day. uMgungundlovu District is one of the ten districts of the KwaZulu-Natal Province, with a population size of 1 052 730, 84.3% of whom are medically uninsured [14]. The three CHCs in the study were Bruntville CHC which is rural and Eastboom CHC and Imbalenhle CHC, both of which are urban [14].

### 2.3. Patient Selection, Enrolment, and Sample Collection

All patients diagnosed with a UTI by the attending clinician or nurse were considered for the study. After an explanation of the purpose of the study, written, informed consent was obtained from each participant. A total of 42, 44 and 57 patients were recruited from Bruntville CHC, Eastboom CHC and Imbalenhle CHC, respectively (no patients with UTI presented at the District Hospital on the day of the study). Both males and females, aged between 18 and 60 years were enrolled. The inclusion criteria considered patients clinically diagnosed with a UTI by nurses or doctors, who voluntarily agreed to participate, signed the consent form, and agreed to provide a mid-stream urine (MSU) sample. The exclusion criterion were patients who had been on antibiotics in the previous three months. 

A questionnaire including demographic data (age, gender, race, and place of residence) was completed. Clinical data were extracted from patient records, and patient information was coded before analysis to maintain confidentiality. All patients clinically diagnosed with UTIs voluntarily consented to participate in the study. Thus, once the consent was obtained, each participant was handed a sterile urine collection cup (BD, Woodmead, South Africa) and the sampling procedure explained. Samples were immediately kept at 4 °C after collection and transported to the laboratory on ice for analysis within eight hours from the time of collection.

### 2.4. Bacterial Isolation and Identification 

Mid-stream urine (MSU) samples were inoculated onto Eosin Methylene Blue (EMB) agar for the putative identification of Gram-negative bacteria [15]. The growth of ≥10^5^ colony forming units (CFU)/mL of colonies of similar morphology was regarded as positive for a UTI [16], while the presence of two different colony types of equal quantity was regarded as potential co-infection. 

Morphologically distinct colonies were selected from each plate and streaked further on EMB plates to obtain pure colonies. Single colonies were again selected and subcultured on nutrient agar plates. All plates were incubated at 37 °C for 18 h. Pure isolates were then subjected to Gram’s staining, catalase and oxidase tests and confirmed using the API 20E test kit (BioMerieux, Midrand, South Africa) according to the manufacturer’s recommendations. *E. coli* ATCC (Amrican Type Culture Collection) 25922 as control. 

### 2.5. Antibiotic Susceptibility Testing

Susceptibility to 17 antibiotics belonging to seven antibiotic classes was determined using the disk diffusion method according to the European Committee on Antimicrobial Susceptibility Testing (EUCAST) guidelines [17]. The antibiotic panel consisted of Penicillins (ampicillin 10 µg; amoxicillin/clavulanic acid 10–20 µg; piperacillin-tazobactam 30–6 µg), Cephalosporins (cefoxitin 30 µg; cefuroxime 30 µg; ceftazidime 10 µg; cefotaxime 5 µg; cefepime 30 µg), Carbapenems (meropenem 10 µg; imipenem 10 µg; ertapenem 10 µg; doripenem 10 µg), Fluoroquinolones (ciprofloxacin 5 µg), Aminoglycosides (gentamycin 10 µg; amikacin 30 µg), Tetracyclines (tigecycline 15 µg), and Sulphonamides (cotrimoxazole 1.25–23.75 µg). Results were interpreted according to the EUCAST breakpoints version 7.1 [17]. *E. coli* ATCC 25922 served as a control. 

Phenotypic identification of β-lactamase-mediated resistance was performed using Rosco kits (ROSCO Diagnostica A/S, Taastrup, Denmark) on Mueller-Hinton agar (MHA) according to the manufacturer’s recommendations. *E. coli* ATCC 25922 was used as a control. 

### 2.6. Molecular Conformation of Isolates and Detection of Antibiotic Resistance Genes

Single colonies were picked from nutrient agar plates and DNA was extracted using the GeneJET Genomic DNA Purification Kit (Thermo Scientific, Johannesburg, South Africa) according to the manufacturer’s instructions. The extracted DNA was used as template for the PCR assays. All PCR assays were performed in a T100 Thermal Cycler (Bio-Rad, Johannesburg, South Africa). The Master Mix and primers were purchased from Inqaba Biotech, Pretoria, South Africa.

The *papC* gene was amplified to classify the isolates as UPEC [18]. The reaction was performed in a final volume of 20 µL made up of 10 µL of Dream Taq PCR Master Mix, 5 µL of nuclease-free water, 0.5 µL of each primer (final concentration 0.5 µM) and 4 µL of template DNA. The optimized cycling conditions comprised of a preliminary activation for 2 minutes at 94 °C, followed by 30 cycles of denaturation at 94 °C for 1 min, annealing for 1 min and elongation at 72 °C for 5 min.

Two separate multiplex PCRs were used for the detection of resistance genes in this study, viz., *bla_TEM_/bla_SHV_*-like multiplex PCR and *bla*_CTX-M_ multiplex PCR (group 1, 2, and 9) [19]. All assays were carried out in a total reaction volume of 50 μL containing 2 μL of template DNA, primer volumes as indicated in Table A1 and 25 μL of One Taq^®^ 2X Master Mix. Nuclease-free water was added to make up the volume to 50 μL. PCR conditions were optimized as follows: 94 °C for 30 seconds, 35 cycles of 30 s of denaturation at 94 °C, 1 min of annealing, 1 min of extension at 68 °C and a final elongation of 5 min at 68 °C. 

PCR amplification of the *qnr*A, *qnr*B, *qnr*S, *gyr*A, *par*C, *aac(6’)-Ib-cr*, and *qep*A genes was done in a final volume of 25 μl after nuclease-free water was added, containing 12.5 μL of One Taq^®^ PCR Master Mix, 6.5 μl of nuclease-free water, forward and reverse primers, and 2 μL template DNA. The cycle comprised of an activation for 30 s at 94 °C, followed by 35 cycles of denaturation at 94 °C for 30 s, annealing for 45 s and elongation at 68 °C for 7 min. 

The annealing temperatures, primer volumes, and sequences are described in Appendix A. All reaction included a no template control (reaction mixture void of DNA) and positive control (template DNA from previously characterized inhouse isolates). The PCR products were loaded onto a 1.0% (w/v) agarose gels stained with SYBR green and subjected to electrophoresis at 100V for 60 minutes using 1× TBE buffer. Amplification products were visualized by UV transillumination using the Bio-Rad molecular imager, Gel Doc XR+ (Bio-Rad, USA) [19]. 

### 2.7. Determination of Clonal Relatedness of Isolates

The clonal relatedness of isolates was ascertained by the Enterobacterial Repetitive Intergenic Consensus (ERIC)-PCR [20]. This was conducted in a total reaction volume of 10 μL, which contained 2 μL of template DNA and 0.1 μL of 100 μM primers ERIC 1 and ERIC 2 (Appendix A) and 5 μL of DreamTaq Green PCR Master Mix (Thermo Scientific, USA). PCR conditions were as follows: 94 °C for 7 min, 30 cycles of 30s of denaturation at 94 °C, 1 min of annealing at 50 °C, 8 min of extension at 65 °C and a final elongation of 16 min at 65 °C, in T100 Thermal Cycler (Bio-Rad, USA) [21]. The ERIC-PCR products were loaded onto a 1.0% (w/v) agarose gel stained with SYBR green and subjected to electrophoresis at 80V using a 1× TAE buffer for 3 h. Gels were visualized and imaged using the DigiGenius gel documentation system (Vacutec, Johannesburg, South Africa) for further analysis. 

The BioNumerics software version 7.6 (Applied Maths, TX, USA) was used for the analysis of the digitalized ERIC profiles. Quick-Load^®^ 1 kb DNA molecular weight marker (Inqaba Biotec, Pretoria, South Africa) was used to normalize the DNA fragments obtained from the ERIC-PCR assay. The similarity between isolates was determined using the Jaccard coefficient. Dendrograms were constructed based on the average similarities of the matrix using the Unweighted Pair-Group Method with Arithmetic mean (UPGMA) algorithm. Optimization and band tolerance were set at 1%, and 60% similarity cut off was used to define clusters.

## 3. Results

### 3.1. Prevalence of UTIs in Participants

A total of 143 participants were recruited for this study, all of whom provided urine samples. Twenty-eight (28) of these samples were positive for culture, giving a UTI prevalence of 19.6%. The distribution of the positive samples was 13, 9, and 6 patients from Imbalenhle, Eastboom and Bruntville CHCs, respectively. Twenty-five percent of the positive samples (7/28) were from males, and 75% (21/28) were from females. Only 14% (3/21) of the women that tested positive for a UTI was pregnant. Most of the patients (53.6%) were in the 18–28 age group while the age group 39–48 recorded the lowest number of patients (17.8%). 

### 3.2. Etiology of UTI in the Sampled Participants

A total of 32 pure bacterial isolates were obtained from the 28 UTI-positive participants. These bacterial isolates were identified as *E. coli* (26; 81.25%), *K. pneumonia* (2; 6.25%), *Citrobacter koseri* (2; 6.25%), *Enterobacter cloacae* (1; 3.125%) and *Bordetella* spp. (1; 3.125%). Co-infection was recorded in four (14.29%) patients. Also, all the 26 (100%) *E. coli* isolates were positive for the *pap*C gene, which codes for proteins that are involved in the P fimbriae assemble in UPEC. 

### 3.3. Antibiotic Resistance Profile of Isolates

**Figure 1:**
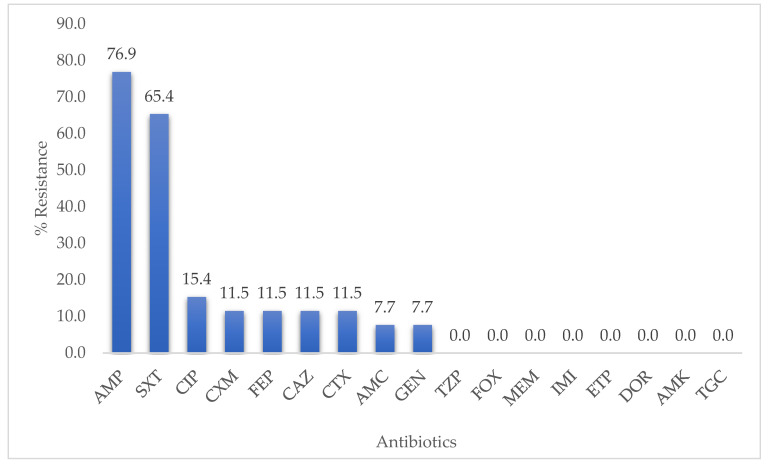
Overall percentage resistance to tested antibiotics. AMP = Ampicillin, SXT = Trimethoprim-sulfamethoxazole, CIP = Ciprofloxacin, CXM = Cefuroxime, FEP = Cefepime, CAZ = Ceftazidime, CTX = Cefotaxime, AMC = Amoxicillin/clavulanate, GEN = Gentamycin, TZP = Piperacillin-tazobactam, FOX = Cefoxitin, MEM = Meropenem, IMI = Imipenem, ETP = Ertapenem, DOR = Doripenem, AMK = Amikacin, TGC = Tigecycline.

All the 26 *E. coli* isolates were further tested for their susceptibility to 17 antibiotics. Twenty-two (22; 84.62%) of these isolates were resistant to at least one of the antibiotics tested in the current study. The highest resistance was observed to ampicillin 20 (76.92%) while the lowest resistance (2; 7.14%) was to amoxicillin/clavulanic acid and gentamycin. Four isolates were susceptible to all antibiotics, and no isolate was resistant to piperacillin-tazobactam, cefoxitin, meropenem, imipenem, ertapenem, doripenem, amikacin and tigecycline (Figure 1). The Rosco test for ESBLs was positive for 3 (10.71%) of the *E. coli* isolates. 

**Figure 2:**
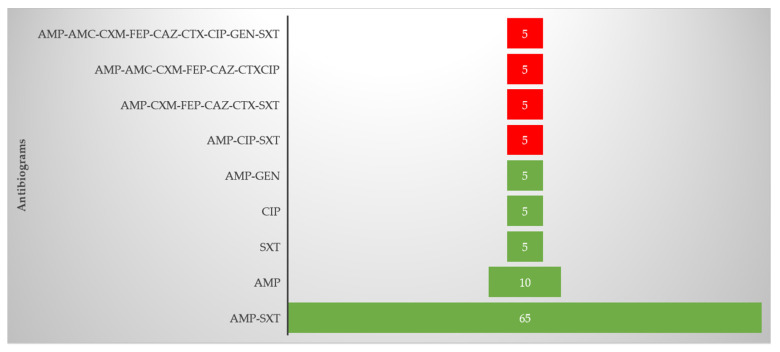
Antibiograms of the resistant isolates. Numbers represent percentage occurrence. The red color denotes the multidrug-resistant isolates. AMP = Ampicillin, SXT = Trimethoprim-sulfamethoxazole, CIP = Ciprofloxacin, CXM = Cefuroxime, FEP = Cefepime, CAZ = Ceftazidime, CTX = Cefotaxime, AMC = Amoxicillin/clavulanate, GEN = Gentamycin.

The resistant isolates displayed nine different antibiograms (Figure 2). The most abundant antibiogram was AMP-SXT, while 20% of the isolates were resistant to only one antibiotic. Four of the isolates were multidrug-resistant (showing resistance to at least three classes of antibiotics), with one isolate showing resistance to up to nine of the 17 antibiotics tested.

### 3.4. Detection of Antibiotic Resistance Genes

All the three (100%) isolates that showed ESBL activity were positive for the *CTX-M* gene, while one (33%) was positive for the *TEM*. None of the isolates harbored the *SHV* gene. The *qnrA*, *qnrB*, *qnrS*, *qepA*, *gyrA*, *parC* and aa*c(6’)-Ib-cr* genes that confer resistance to fluoroquinolones were detected at different percentages in the isolates (Table 1). 

### 3.5. Determination of Clonality Through ERIC-PCR 

**Figure 3:**
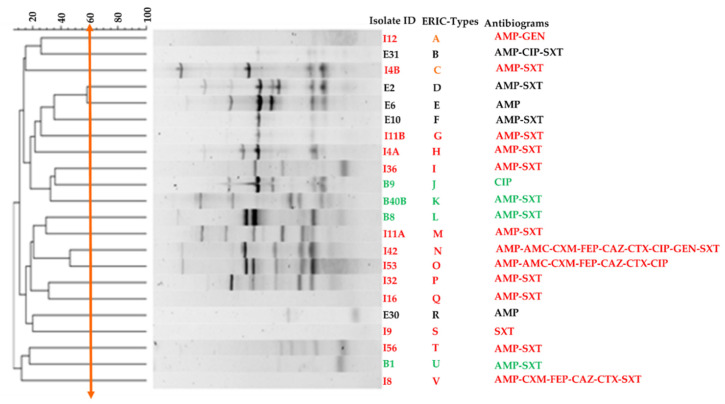
Dendrogram showing cluster analysis of the antibiotic-resistant UPEC strains isolated from urine samples. The solid orange line represents ERIC_Types cut off. The colors on the annotations represent the isolate sources: Bruntville (green), Eastboom (black), Imbalenhle (red).

The ERIC-PCR fingerprint profiles of the 22 antibiotic-resistant UPEC isolates tested are shown in Figure 3. The isolates exhibited differences in banding patterns, placing each isolate on a distinct cluster, based on a 60% similarity index. 

## 4. Discussion

### 4.1. Prevalence of UTIs in Pparticipants

Urinary tract infections are the second most diagnosed bacterial infections in community medicine worldwide [22]. In the current study, 143 patients were clinically diagnosed with UTI by the nurses and clinicians. However, only 28 of these patients were positive for UTIs of Gram-negative bacterial etiology based on further urine analysis, giving a prevalence of 19.6%. Assuming minimal Gram-positive etiology in UTIs, this study showed a potential over-diagnosis of UTIs based on clinical assessments, mostly by primary healthcare nurses in CHCs. Such over-misdiagnosis would result in the unnecessary administration of empirical antibiotics, leading to several adverse consequences such as an increase in antibiotic resistance as well as cost to the hospitals and patients [23]. 

Although UTIs affect people of all genders and ages, certain predisposing factors such as being elderly, pregnancy status and other predisposing conditions, among others, could increase the risk of infection [24]. Nevertheless, the majority of the patients with UTIs in the current study were females (75%), which was expected as UTIs are more common in females mainly because of their anatomical and physiological characteristics [25]. Also, a small number of pregnant women with UTIs (14%) was ecorded in the present study and this could be explained by the fact that antenatal clinics functioned separately from the primary healthcare clinics at the CHCs investigated. UTIs were most common in the 18 to 28-year age group which could be due to higher sexual activities, as it is reported that highest sexual activities was observed in women between the ages of 18 and 39 [26]. Frequent intercourse has been listed among risk factors for developing a UTI [27], while an association between frequency of intercourse and the chances of developing a UTI has been demonstrated [28,29]. It should, however, be noted that while such associations have been reported in previous studies, other studies have reported contrary findings [30], therefore, suggesting the need for further studies to ascertain the link between frequent sexual activities and the prevalence of UTIs. Also, the higher prevalence recorded in the 18 to 28-year age group in the current study should not be over generalized to the entire population, as this prevalence could have been influenced by the fact that over 50% of the participants were in this age group. Further epidemiological studies, involving a larger sample size and conducted over a longer period, could eliminate sample bias, and give a better representation of the prevalence of UTIs in the general population.

### 4.2. Etiology of UTI in the Sampled Participants

*Escherichia coli* is the most common occurring uropathogen in UTIs [31,32,33,34], accounting for 75–95% of all CA-UTIs reported globally [33]. These reports corroborate the finding of the current study in which over 80% of isolates obtained from the cultured urine samples were *E. coli*. These isolates were all positive for the *pap*C gene, characteristic of UPEC [35]. The *pap*C gene is a significant determinant of the progression of infection caused by UPEC as it codes for proteins involved in the assembling of the P fimbriae, which is a surface virulence factor of *E. coli* [36]. Although UPEC was the most isolated uropathogen in the present study, the other organisms identified have also been implicated in UTIs. For example, *K. pneumonia* was reported in cases of CA-UTIs in Morocco [12] and Portugal [2]. Similarly, *Citrobacter koseri* [37] and *Enterobacter cloacae* [38] have been implicated in UTIs, although in hospital settings and patients with other predisposing complications. It should, however, be noted that the current study targeted *E. coli*, and only used EMB agar, which could have prevented the isolation of other bacterial species.

### 4.3. Antibiotic Resistance Profile of Isolates

Many factors affect the choice of first-line antibiotics for a specific infection. These include susceptibility profiles of the etiologic agents or the therapeutic effectiveness of the drug, side effects of the antibiotics, the financial cost to both the patient and the healthcare center, and the ability to develop new antibiotics to overcome selection pressure caused by antibiotic resistance [33]. According to the Standard Treatment Guidelines (STG) and Essential Medicine List (EML) for South Africa, the first line of antibiotics for the treatment of UTIs are ciprofloxacin, amoxicillin/clavulanic acid, and nitrofurantoin [39]. The community healthcare centers that were investigated prescribed ciprofloxacin and amoxicillin/clavulanic acid for infections based on the country’s guidelines. 

Despite the establishment of proper steps for antibiotic prescription, antibiotics still become less effective the more they are prescribed [40]. In the current study, the isolated UPEC strains had developed considerable resistance to the STGs-recommended antibiotics as there was 15.4% resistance to ciprofloxacin and 7.7% to amoxicillin/clavulanic acid (Figure 1). These results correlate with the results obtained from previously reported studies in South Africa [33,41]. The observed resistance could have been fueled by the over-diagnosis of UTIs that could have further led to the indiscriminate and undeserved prescription of these drugs to patients who did not require them. This, therefore, calls for the need to improve on stewardship and encourage a revision of prescribing habits [42]. To achieve this, it would be beneficial to combine clinical observations with laboratory diagnosis, especially in uncomplicated UTIs that may not necessarily require immediate commencement of treatment. Furthermore, the results of the current study, and those of previous ones conducted in South Africa, suggest that the standard treatment guidelines (STGs) for UTIs at CHC level need to be revised to ensure the effective treatment of patients and for the preservation of antibiotics.

Despite the high resistance to some of the antibiotics tested and to the STGs-recommended ones, the isolates were 100% susceptible to piperacillin-tazobactam, cefoxitin, meropenem, imipenem, ertapenem, doripenem, amikacin and tigecycline, suggesting that these drugs could be tested further as alternatives for treating CA-UTIs in the rural and urban communities.

The treatment of CA-UTIs is further exacerbated by the fact that many uropathogens, including UPEC strains, have become multidrug-resistant [43]. In the current study, four (18.2%) of the resistant isolates were multidrug-resistant, with one isolate being resistant to up to 9 of the 17 antibiotics tested (Figure 2). Of all the resistant isolates, ESBLs were identified in 3 (13.6%) isolates whereas fluoroquinolone resistance was identified in 4 (18.2%). The ESBL-positive isolates harbored the *TEM* and *CTX-M* groups 1, 2, and 9 genes, with *CTX-M* being the most common gene identified. ESBLs cause significant resistance to cephalosporins, penicillin, and aztreonam by hydrolyzing the antibiotics [43], which explains the resistance to ampicillin, cefuroxime, cefepime, ceftazidime, and cefotaxime, observed in all the ESBL-positive isolates in this study. 

The three multidrug-resistant isolates that produced ESBLs were also resistant to fluoroquinolones and harbored more than one type of fluoroquinolone resistance gene, both chromosomal and plasmid-mediated. These results correlate with the results of a South African study conducted in 2016 in which similar fluoroquinolone resistance genes were identified [44]. 

The high Trimethoprim-sulfamethoxazole resistance (65.4%) was expected because this drug is extensively used at community level as it is cheap, administered orally, readily available at the community healthcare centers and is used for infections other than UTIs; it is also extensively used for *Pneumocystis carinii* pneumonia (PCP) prophylaxis as a result of the high HIV and AIDS prevalence in South Africa [45]. Similar high resistance rates have previously been reported in isolates recovered from CA-UTI patients in eastern [46].

### 4.4. Clonal Relatedness of Isolates

The results of the ERIC-PCR showed a high diversity of the isolates, at a cut-off value of 60% (Figure 3). This could suggest the circulation of diverse CA-UTI-associated UPEC clones within South African communities. However, these results should be interpreted with care given the study design (point prevalence) and the small number of isolates that were obtained. Also, the health district is stratified into CHCs and District Hospitals (DHs). This means that patients would typically attend the closest health facility in their communities, and only get referred to the DHs in cases of complication or treatment failure at the CHC level. This could prevent the spread of bacterial strains between the various communities. Thus, the results presented in the current study may not be generalized as only CHCs were investigated. It is, therefore, suggested that further studies be conducted at larger scales, involving the secondary and tertiary hospitals and a larger number of isolates, to have a better picture of the spread. Despite the difference in the clonal patterns, the most identified antibiogram, APM-SXT, was noted in all three CHCs, indicating the potential uniformity in the use of antibiotics in the three communities. Also, the high diversity in the resistance patterns, even in those from the same community, suggests that different resistance mechanisms could be involved, requiring more extensive studies to elucidate these.

## 5. Conclusions

The prevalence of CA-UTIs of Gram-negative etiology among adults 18–60 years of age in the uMgungundlovu District was 19.6%, with UPEC accounting for 81.3% of all the pathogens isolated. Up to 84.6% of these isolates were resistant to at least one of the antibiotics tested. A significant percentage of isolates were resistant to the South African recoomended antibiotics for the treatment of UTIs. Some isolates were also multidrug-resistant and harbored genes that confered resistance to estended-spectrum beta-lactams and fluoroquinolones. Isolates displayed extensive clonal diveresity based on the ERIC-PCR analyses. The results obtained in this study suggest that better diagnostic measures need to be implemented to prevent over prescription of antibiotics. They also suggest that there is need to revise the current treatment guidelines, to ensure effective treatment of CA-UTIs in South Africa. Further studies invloving more participants, more isolates, and advanced techniques such as whole-genome sequencing are required to better understand the antimicrobial susceptibility and molecular characterisitics of UPEC strains involved in CA-UTIs in South African rural and urban communities, thus informing the choice of antibiotics for use in the treatment of these infections.

## Figures and Tables

**Table 1:** Resistance (ESBL and Fluoroquinolone) Genes Detected in *E. coli.*

Isolate ID	β-Lactam Resistance Genes			Fluoroquinolone Resistance Genes					
	*TEM*	*SHV*	*CTX-M* (Group 1,2,9)	*gyr*A	*qnr*A	*qnr*B	*qnr*S	*aac (6’)-Ib-cr*	*qep*A
I8	−	−	1,2,9	−	−	−	−	−	−
I42	+	−	1,2	+	+	−	+	+	−
I53	−	−	1,2	−	+	−	+	+	−
B9	−	−	−	+	−	−	−	−	−
E31	−	−	−	+	−	+	−	+	+

## Appendix A

**Table A1:** Primers, Primer Concentrations and Annealing Temperatures Used for the Molecular Characterization of the *E. coli* Isolates in this Study.

Gene	Sequences 5′ to 3′	Primer (µL)	Annealing Temperature (°C)	Reference
*TEM*	F-CATTTCCGTGTCGCCTTATTC	2	60	[47]
	R-CGTTCATCCATAGTTGCCTGAC	2		
*SHV*	F-AGCCGCTTGAGCAAATTAAAC	2	60	[47]
	R-ATCCCGCAGATAAATCACCAC	2		
*bla_CTX-M-1_*	F-TTAGGAARTGTGCCGCTGYA	2	60	[47]
	R-CGATATCGTTGGTGGTRCCAT	1		
*bla_CTX-M-2_*	F-CGTTAACGGCACGATGAC	1	60	[47]
	R-CGATATCGTTGGTGGTRCCAT	1		
*bla_CTX-M-9_*	F-TCAAGCCTGCCGATCTGGT	2	60	[47]
	R-TGATTCTCGCCGCTGAAG	2		
*qnr*A	F-AGAGGATTTCTCACGCCAGG	2	55	[46]
	R-TGCCAGGCACAGATCTTGAC	2		
*qnr*B	F-GGAATCGAAATTCGCCACTG	2	55	[46]
	R-TTTGCCGTTCGCCAGTCGAA	2		
*qnr*S	F-CACTTTGATGTCGCAGAT	2	55	[48]
	R-CAACATACCCAGTGCTT	2		
*aac(6’)-Ib-cr*	F-GATGCTCTATGGGTGGCTAA	2	55	[49]
	R-GGTCCGTTTGGATCTTGGTGA	2		
*qep*A	F-CCGATGACGAAGCACAGGG	2	55	[49]
	R-CTACGGGCTCAAGCAGTTGG	2		
*gyr*A	F-GGTACACCGTCGCGTACTTT	2	55	[50]
	R-CAACGAAATCGACCGTCTCT	2		
*par*C	F-GCCTTGCGCTACATGAATTT	2	55	[50]
	R-ACCATCAACCAGCGGATAAC	2		
*pap*C	F-GACGGCTGTACTGCAGGGTGGCG	0.5	55	[18]
	R-ATATCCTTTCTGCAGGGATGCAATA	0.5		
ERIC	ERIC1-ATGTAAGCTCCTGGGGATTCAC	0.1	50	[21]
	ERIC2-AAGTAAGTGACTGGGGTGAGCG	0.1		
